# Proinflammatory Factors Mediate Paclitaxel-Induced Impairment of Learning and Memory

**DOI:** 10.1155/2018/3941840

**Published:** 2018-02-22

**Authors:** Zhao Li, Shuang Zhao, Hai-Lin Zhang, Peng Liu, Fei-Fei Liu, Yue-Xian Guo, Xiu-Li Wang

**Affiliations:** ^1^Department of Anesthesiology, The Third Hospital of Hebei Medical University, Shijiazhuang, Hebei 050011, China; ^2^Department of Pharmacology, Hebei Medical University, Shijiazhuang 050017, China; ^3^Department of Urology, The Third Hospital of Hebei Medical University, Shijiazhuang, Hebei 050011, China

## Abstract

The chemotherapeutic agent paclitaxel is widely used for cancer treatment. Paclitaxel treatment impairs learning and memory function, a side effect that reduces the quality of life of cancer survivors. However, the neural mechanisms underlying paclitaxel-induced impairment of learning and memory remain unclear. Paclitaxel treatment leads to proinflammatory factor release and neuronal apoptosis. Thus, we hypothesized that paclitaxel impairs learning and memory function through proinflammatory factor-induced neuronal apoptosis. Neuronal apoptosis was assessed by TUNEL assay in the hippocampus. Protein expression levels of tumor necrosis factor-*α* (TNF-*α*) and interleukin-1*β* (IL-1*β*) in the hippocampus tissue were analyzed by Western blot assay. Spatial learning and memory function were determined by using the Morris water maze (MWM) test. Paclitaxel treatment significantly increased the escape latencies and decreased the number of crossing in the MWM test. Furthermore, paclitaxel significantly increased the number of TUNEL-positive neurons in the hippocampus. Also, paclitaxel treatment increased the expression levels of TNF-*α* and IL-1*β* in the hippocampus tissue. In addition, the TNF-*α* synthesis inhibitor thalidomide significantly attenuated the number of paclitaxel-induced TUNEL-positive neurons in the hippocampus and restored the impaired spatial learning and memory function in paclitaxel-treated rats. These data suggest that TNF-*α* is critically involved in the paclitaxel-induced impairment of learning and memory function.

## 1. Introduction

The chemotherapeutic agent paclitaxel is widely used to treat patients with breast, ovarian, and lung cancers [1–5]. By binding to *β*-tubulin within the microtubules, paclitaxel stabilizes the microtubule lattice to suppress depolymerization and dynamic instability [6]. Paclitaxel treatment induces mild to moderate cognitive impairment known as “chemobrain” in some cancer survivors [7–12]. Compared with the 8% incidence rate of cognitive concerns in participants without a prior cancer diagnosis, an approximately 14% incidence rate of cancer survivors with memory problems represents a 40% increase in cancer survivors who have cognitive concerns [13]. However, the mechanisms underlying paclitaxel-induced neurological dysfunction remain unclear.

Previous studies have shown that the neuropathy of paclitaxel is limited to peripheral sensory nerves through affecting axonal degeneration in sensory nerves [14–16] because paclitaxel does not cross the blood-brain barrier (BBB) [17]. However, by using positron emission tomography (PET), radiolabeled paclitaxel was detectable in the brain tissues after intravenous administration [18], suggesting that a small amount of paclitaxel may cross the BBB to act on brain tissue. Furthermore, paclitaxel induces apoptosis in neurons through a mechanism distinct from that of nonneuronal cells [19]. In this regard, paclitaxel induces apoptosis through endoplasmic reticulum stress [20]. The hippocampus, cortex, and striatum are the core regions in the limbic system [21] and play critical roles in spatial learning and memory as well as cognitive processes [22, 23]. However, the mechanisms underlying the neurotoxicity of paclitaxel on hippocampus neurons are not clear.

Paclitaxel increases the production of TNF-*α* in macrophages and endothelial cells [24–26] by promoting TNF-*α* gene expression [27]. TNF-*α* is a membrane-integrated proinflammatory cytokine which is produced by activated macrophages and monocytes [28]. TNF-*α* can induce cell death or a cell-protective effect depending on the receptor it binds to TNF-*α* receptor 1 (TNFR1) or 2 (TNFR2) [29, 30]. TNF-*α* preferentially binds to TNFR1 which contains a death-effector domain to induce caspase-8 cleavage and apoptosis [31, 32]. It has been shown that TNF-*α* can initiate apoptosis of neurons [33–35] and oligodendrocytes [36, 37]. Therefore, we hypothesized that neuronal apoptosis caused by proinflammatory factors contributes to paclitaxel-induced impairment of neurological function.

## 2. Materials and Methods

Male Sprague-Dawley (SD) rats (6–8 weeks old, 200–300 g) were used in this study. The experimental protocol was approved by the Use Committee of the Hebei Medical University according to the guidelines of using animals (K2016-020-3). All efforts were made to minimize both the suffering and the number of rats used. The rats were numbered during the study. The number identity of the rats was revealed during data analysis. Quantitative data were analyzed by staff blinded to group-identifying information.

### 2.1. Learning and Memory Tests Using Morris Water Maze

Male SD rats were randomly assigned into experimental groups for MWM test following randomized numbers generated by the GraphPad software. The sample size and power were determined by using according to the data obtained from our pilot studies by using the SPSS Statistics analysis software. The rats were closely observed by staff from the lab and animal facility for fatal signals and any abnormal behaviors such as pain, distress, and infection, as described in the animal protocol. The MWM testing experiments were performed by staff blinded to treatment conditions. The water maze container filled with opaque water was 150 cm in diameter and 50 cm in height of the wall. The water level was 32 cm in height. The escape platform was 30 cm in height and 15 cm in diameter. The MWM tests were performed before and every 4 days after paclitaxel and vehicle treatment (an average of values of 4 tests during the testing day). The escape latency and the number of crossings over the platform location were analyzed and plotted.

### 2.2. Paclitaxel Treatment

**Figure 1:**
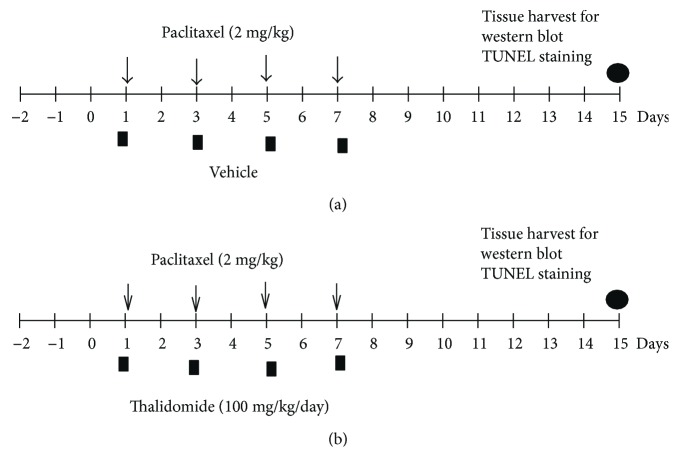
Experimental procedure. Treatment schedule for paclitaxel or vehicle (a) and paclitaxel and thalidomide (b) in the different experimental groups.

Paclitaxel was dissolved in a vehicle solution containing a mixture of saline and 10% Cremophor EL, a derivative of castor oil and ethylene oxide, which is clinically used for paclitaxel injection. Vehicle or paclitaxel solution was administered intraperitoneally (i.p.) in a dosage of 2 ml/kg/day. After habituation to the test environment and baseline measurement of behavioral tests, paclitaxel or vehicle was injected administered to these 2 groups of rats on 4 consequent days (days 1, 3, 5, and 7) with paclitaxel at a dose of 2 mg/kg to reach a final cumulative paclitaxel dose of 8 mg/kg [38]. TNF-*α* inhibitor thalidomide was administered orally (100 mg/kg/day) for 4 days during paclitaxel treatment [39]. The treatment schedule is illustrated in Figure 1.

### 2.3. Western Blot Assay

The Western blot analysis was performed by staff blinded to treatment conditions. Under deep anesthesia by intraperitoneal injection of pentobarbital (60 mg/kg), the rat was decapitated and the hippocampus tissue was removed and homogenized in RIPA lysis buffer containing proteinase F. An equal amount of protein (60 *μ*g) was loaded into each lane, separated electrophoretically by SDS-PAGE and electroblotted onto PVDF membranes. After blocking for 1 h in 5% nonfat dry milk, the membrane was incubated with anti-TNF-*α* and anti-IL-1*β* and anti-GAPDH (Santa Cruz) at 4°C overnight. The membrane was rinsed and incubated with horseradish peroxidase-conjugated goat anti-rabbit secondary antibody at 1 : 4000 dilutions for 3 h at 37°C. The membrane was developed by using an enhanced chemiluminescence kit according to the manufacturer's instruction. The blotting was captured digitally, and the intensity of the band was quantified by the Kodak EDAS 120 system and Quantity One 4.6 software. The density of each band was normalized to GAPDH expression.

### 2.4. TUNEL Assay

For visualization of neuronal apoptosis in the hippocampus, we performed terminal deoxynucleotidyl transferase dUTP nick end labeling (TUNEL) assay to assess apoptotic cell death in rats treated by vehicle, paclitaxel, thalidomide, and paclitaxel and thalidomide. In brief, the rats were anesthetized and perfused with 200 ml of 0.9% normal saline with 0.1% of heparin, followed by 200 ml PBS containing 4% paraformaldehyde. Brains were removed and placed in 4% paraformaldehyde to postfix for 24 h at room temperature. Then, the brains were dehydrated and sectioned into brain slices at a thickness of 16 *μ*m. An In Situ Cell Death Detection Kit (TMR Green; Roche Applied Sciences, Indianapolis, IN) was used to determine the number of apoptotic cells. Ten sections randomly selected from each group were fixed in ethanol-acetic acid at −20°C. The sections were incubated with proteinase K, rinsed, and incubated with 3% H_2_O_2_ at room temperature. Then, 0.5% Triton X-100 was used to permeabilize the cell membrane followed by incubation with the TUNEL reaction mixture (6 *μ*l per section) at 37°C. The sections were then visualized using Converter-POD with 0.02% 3,3′-diaminobenzidine (DAB; 100 *μ*l/section) at room temperature. Then, the sections were counterstained with hematoxylin for 30 seconds and washed again with running water for 10 minutes. Cell counting was performed in the CA1 subfield of the dorsal hippocampus. Cells with brown nuclei were considered apoptotic and were analyzed by using the Image-Pro 6.0 software (Media Cybernetics Inc., Rockville, MD, USA). The number of TUNEL-positive cells was quantified under ×400 magnification, and the density of stained cells was presented as cells per square millimeter.

### 2.5. Statistical Analysis

All data were expressed as mean ± SEM, and the data were analyzed by the SPSS 13.0 software (SPSS Inc., Chicago, IL, USA). For comparisons of more than 2 groups, the repeated measures ANOVA with Dunnett's post hoc test or one-way ANOVA with Bonferroni's post hoc test was performed to compare responses within or between experimental groups (GraphPad Prism 6). *P* < 0.05 was considered statistically significant.

## 3. Results

### 3.1. Paclitaxel Impairs Spatial Memory in the MWM Test

**Figure 2:**
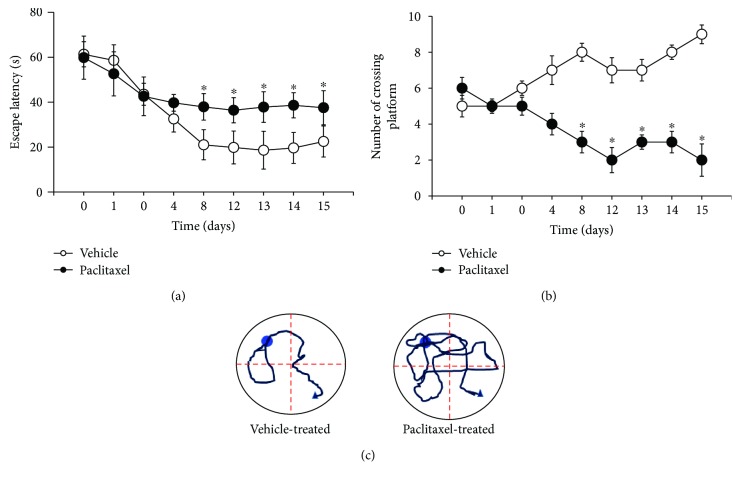
Paclitaxel impairs spatial learning and memory in the Morris water maze test. Summary data showing the escape latency (a) and the number of crossings (b) over the platform during a series of testing before and after paclitaxel or vehicle treatment. Representative swimming paths on the 12th day after paclitaxel or vehicle treatment (c). Data are expressed as mean ± SEM. ^∗^*P* < 0.05 compared with the vehicle-treated group.

It has been shown that acute application of paclitaxel impairs cognitive function in mice [40]. Thus, we first determined the cognitive function in rats subjected to chronic paclitaxel treatment by performing the MWM test. The escape latencies and the number of crossings over the hidden escape platform beneath the water were significantly decreased in the later trails following the initial trail and reached a stable level at trial numbers 5 to 9 in vehicle-treated rats (*n* = 15). Another group of rats (*n* = 15) was treated with paclitaxel (2.0 mg/kg/day for 4 days, i.p.). Paclitaxel treatment significantly increased the duration of escape latencies and the number of crossings over the platform compared with vehicle-treated rats (Figures 2(a) and 2(b)). These data suggest that chronic paclitaxel impairs spatial learning and memory.

### 3.2. Paclitaxel-Induced Hippocampal Neuronal Apoptosis

**Figure 3:**
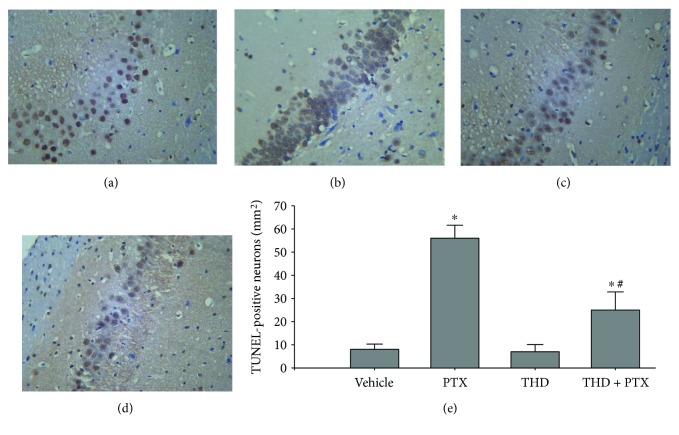
Cell apoptosis in the hippocampal CA1 region of rats treated with paclitaxel. (a–d) TUNEL staining was used to observe differences in apoptosis (×200). Paclitaxel treatment significantly increased TUNEL-positive cells (b) while thalidomide significantly decreased TUNEL-positive cells in paclitaxel-treated rats (d). (e) Quantitative analysis of TUNEL-positive neurons per square millimeter in each group. No significant difference in the number of TUNEL-positive cells was observed between the vehicle- and thalidomide-treated rats. Data are expressed as mean ± SEM (*n* = 10) and analyzed using one-way analysis of variance and Dunnett's post hoc test. ^∗^*P* < 0.05 compared with the vehicle group. ^#^*P* < 0.05 compared with the PTX group. Vehicle: vehicle-treated group; PTX: paclitaxel-treated group; THD: thalidomide-treated group; THD + PTX: paclitaxel- and thalidomide-treated group; TUNEL: terminal deoxynucleotidyl transferase dUTP nick end labeling.

We performed TUNEL staining to evaluate cell apoptosis in the hippocampus. Paclitaxel treatment significantly increased the number of TUNEL-positive cells in the hippocampus compared with vehicle-treated rats (*P* < 0.05, Figure 3). Treatment with thalidomide, a TNF-*α* synthesis inhibitor, did not change the number of TUNEL-positive cells in vehicle-treated rats. However, thalidomide treatment significantly reduced the number of TUNEL-positive cells in paclitaxel-treated rats in the hippocampal CA1 region (Figure 3). No significant difference was detected in the number of TUNEL-positive cells between the vehicle-treated and thalidomide-treated rats. These results suggest that paclitaxel induces cell apoptosis in the hippocampus through TNF-*α*.

### 3.3. Paclitaxel Increases Expression Levels of Proinflammatory Cytokines

**Figure 4:**
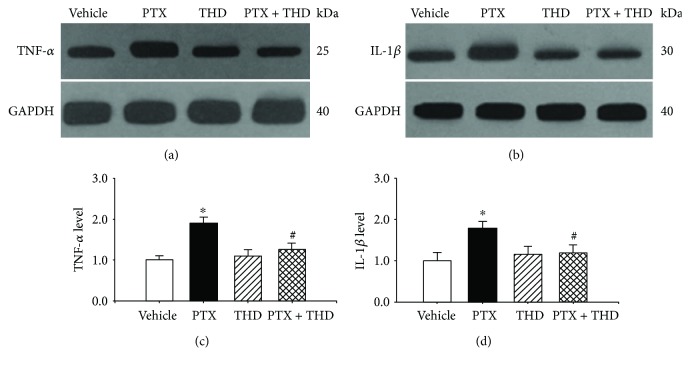
Paclitaxel increased the expression levels of TNF-*α* and IL-1*β*. Representative gel images (a and b) and summary data (c and d) show the TNF-*α* (a) and IL-1*β* (b) protein expression levels in the hippocampus tissue from rats treated with vehicle, paclitaxel, thalidomide, and paclitaxel plus thalidomide (*n* = 6 rats in each group). Data in (c) and (d) are expressed as mean ± SEM. ^∗^*P* < 0.05 compared with the vehicle group. ^#^*P* < 0.05 compared with the PTX group. PTX: paclitaxel; THD: thalidomide.

Then, the expression levels of proinflammatory cytokines TNF-*α* and IL-1*β* in the hippocampus were determined in the vehicle- and paclitaxel-treated rats. We performed Western immunoblotting analysis by using antibodies against TNF-*α* and IL-1*β* in hippocampal tissues obtained from rats treated with vehicle, paclitaxel, and paclitaxel with thalidomide (TNF-*α* inhibitor, 100 mg/kg/day). Western immunoblotting showed a single band for TNF-*α* or IL-1*β*. Paclitaxel treatment significantly increased the band density for both TNF-*α* and IL-1*β* (Figure 4). However, thalidomide application reduced the TNF-*α* and IL-1*β* expression levels in paclitaxel-treated rats. Thalidomide treatment had no effect on the expression levels in vehicle-treated rats.

### 3.4. Inhibition of TNF-*α* Rescued the Impairment of Spatial Learning and Memory Induced by Paclitaxel

**Figure 5:**
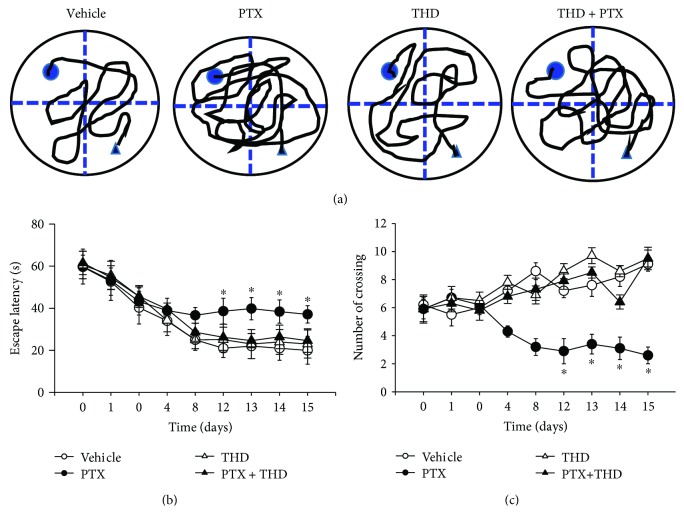
The TNF-*α* synthesis inhibitor thalidomide restored paclitaxel-induced impairment of spatial learning and memory. Representative swimming paths (a) on the 12th day after vehicle, paclitaxel, thalidomide, and paclitaxel plus thalidomide treatment. Summary data showing the escape latency for finding the platform (b) and the number of crossings over the platform (c) during a series of testing in the group of rats receiving vehicle, paclitaxel, and paclitaxel plus thalidomide. Data are expressed as mean ± SEM. ^∗^*P* < 0.05 compared with the vehicle group. PTX: paclitaxel; THD: thalidomide.

Next, we determined the effect of the TNF-*α* inhibitor on spatial learning and memory in rats subjected to chronic paclitaxel treatment. The rats in each group received the injection of vehicle, paclitaxel (2 mg/kg/day, i.p.) for 4 days, and paclitaxel plus oral administration of thalidomide (100 mg/kg/day) for 4 days. During the MWM test, the rats in each group showed a daily improvement in their ability to locate the hidden platform. Paclitaxel treatment significantly increased the escape latencies and the number of crossings compared with vehicle-treated rats (Figure 5). Thalidomide treatment restored the increased escape latencies and the number of crossings in paclitaxel-treated rats, while it had no effect on escape latencies and the number of crossings in vehicle-treated rats. These data suggest that inhibition of TNF-*α* restores paclitaxel-induced impairment of spatial learning and memory.

## 4. Discussion

For the first time, this study demonstrated that chronic systemic administration of paclitaxel impaired spatial learning and memory function through TNF-*α*. Hippocampal cell apoptosis caused by paclitaxel may contribute to the impairment of this neurological function. We found that inhibition of TNF-*α* synthesis restored the impaired learning and memory function in paclitaxel-treated rats. Furthermore, TNF-*α* synthesis inhibition eliminated paclitaxel-induced cell apoptosis in the hippocampus. These findings provide new information about the neurotoxicity of paclitaxel and for developing new therapeutics to treat the side effects of paclitaxel through targeting TNF-*α*.

Paclitaxel is one of the most effective chemotherapeutic agents for the treatment of various types of cancer. By stabilizing microtubules, paclitaxel treatment causes mitochondrial damage and p53-independent cell apoptosis through blocking of the G2/M cell cycle [41–44]. The limitation for the usage of paclitaxel in cancer treatment includes neurotoxicity during paclitaxel therapy [45]. For example, systemic administration of paclitaxel produces painful peripheral neuropathies [46, 47]. We found in this study that paclitaxel significantly increased the escape latency and decreased the number of crossings over the hidden platform. These findings suggest that paclitaxel impairs spatial learning and memory function. Our findings are consistent with commonly reported cognition defects associated with chemotherapy treatment in cancer patients [48, 49]. It has been shown that paclitaxel impairs learning and memory function in rodents [50–52]. However, paclitaxel does not affect all forms of cognition. For example, systemic administration of paclitaxel does not impair spatial and episodic memory in source memory task testing, a radial arm maze procedure that requires the animal to remember the source of information [38]. However, early studies have shown that paclitaxel does not cross the blood-brain barrier (BBB) to affect neurons in the central nervous system [17]. However, later studies using positron emission tomography (PET) found that radiolabeled paclitaxel was detectable in the brain after intravenous administration [18]. Systemic administration of paclitaxel may penetrate at a low rate into the central nervous system to affect cognitive function. Thus, it is possible that paclitaxel may directly cause hippocampal neuronal apoptosis. However, the concentration of paclitaxel in the hippocampus may not be high enough to cause neuronal apoptosis during paclitaxel treatment.

Consistent with previous studies showing that paclitaxel can induce apoptosis of neurons [19, 20], we found that paclitaxel treatment increased the number of TUNEL-positive neurons in the hippocampus. Paclitaxel induces neuronal apoptosis through a mechanism distinct from that in nonneuronal cells. In this regard, by stabilizing microtubules, paclitaxel induces apoptosis in cancer and nonneuronal cells [53, 54]. Paclitaxel induces apoptosis in cortical neurons by a mechanism independent of Bcl-2 phosphorylation [19]. Paclitaxel also induces endoplasmic reticulum stress to cause neuronal apoptosis [20]. We found that paclitaxel induces hippocampal neuronal apoptosis associated with an increase in expression levels of TNF-*α* and IL-1*β* in the hippocampus tissue. One possible mechanism is that paclitaxel activates the p38 MAPK/NF-*κ*B pathway in peripheral macrophages and monocytes. The activation of NF-*κ*B promotes the expression of various proinflammatory genes including TNF-*α*, IL-1*β*, and interleukin-6 (IL-6) [55]. The circulating TNF-*α* released from peripheral macrophages and monocytes increases the permeability of the BBB [56, 57]. Thus, both TNF-*α* and paclitaxel possibly penetrate the BBB to access the central nervous system. Consequently, TNF-*α* activates the NF-*κ*B signaling pathway and inflammatory responses in neural tissues such as microglia, astrocytes, and neurons. It has been shown that sustained activation of the NF-*κ*B-TNF-*α* pathway triggers neuroinflammatory response to promote local neuronal apoptosis [33, 58].

Previous studies have shown that paclitaxel is capable of increasing the expression of IL-1*β* in primary human monocytes, T lymphocytes, human breast cancer cell lines [59], and macrophages [60]. Because paclitaxel led to IL-1*β* synthesis in macrophages at early time points but secreted it at following time points [60], it is likely that TNF-*α* triggers the release of IL-1*β* from macrophages. It should be noted that TNF-*α* is a cytokine produced predominantly by macrophages and it stimulates macrophages to release inflammatory cytokines [61]. This notion is supported by the finding that the TNF-*α* inhibitor also decreased IL-1*β* protein expression levels. This is not a nonspecific effect because the TNF-*α* inhibitor alone did not change the expression level in control rats. It has been shown that a high plasma level of proinflammatory cytokine IL-6 is associated with the occurrence of cognitive impairment in postchemotherapy breast cancer patients [62]. This high level of IL-6 may result from paclitaxel-induced TNF-*α*. However, in this study, we did not determine the role of IL-6 in paclitaxel-induced impairment of learning and memory as well as neuronal apoptosis in the hippocampus. Thus, the role of IL-6 in mediating paclitaxel-induced responses warrants further studies.

In summary, our findings indicate that paclitaxel treatment induces neuronal apoptosis through the upregulation of TNF-*α*. Inhibition of TNF-*α* synthesis with thalidomide rescued paclitaxel-induced impairment of spatial learning and memory function. Our findings identified a novel mechanism underlying paclitaxel-induced impairment of cognitive function. Findings from this study provide new insight into the mechanisms underlying paclitaxel-induced impairment of spatial learning and memory and new targets for the development of novel therapy of neurological dysfunctions during chemotherapy by targeting TNF-*α*.

## Abbreviations

TNF-*α*: Tumor necrosis factor-*α*
IL-1*β*: Interleukin-1*β*
IL-6: Interleukin-6
MWM: Morris water maze.

## Additional Points

*Highlights*. (i) Paclitaxel induces impairment of learning and memory function. (ii) The behavior impairment induced by paclitaxel is associated with an increase in TNF-*α* expression level and apoptotic cells in the hippocampus. (iii) Inhibition of TNF-*α* synthesis recovered the impaired learning and memory function in paclitaxel-treated rats.
